# Polystyrene Magnetic Nanocomposites as Antibiotic Adsorbents

**DOI:** 10.3390/polym12061313

**Published:** 2020-06-09

**Authors:** Leili Mohammadi, Abbas Rahdar, Razieh Khaksefidi, Aliyeh Ghamkhari, Georgios Fytianos, George Z. Kyzas

**Affiliations:** 1 PhD of Environmental Health, Infectious Diseases and Tropical Medicine Research Center, Resistant Tuberculosis Institute, Zahedan University of Medical Sciences, Zahedan 98167-43463, Iran; lailimohamadi@gmail.com; 2 Department of Physics, Faculty of Science, University of Zabol, Zabol 538-98615, Iran; 3 Department of Environmental Health, Zahedan University of Medical Sciences, Zahedan 98167-43463, Iran; r.khaksefidi110@gmail.com; 4 Institute of Polymeric Materials, Faculty of Polymer Engineering, Sahand University of Technology, Tabriz 51335-1996, Iran; aliyeh_ghamkhari@yahoo.com; 5 Department of Chemistry, International Hellenic University, Kavala 65404, Greece; gfytianos@gmail.com

**Keywords:** ciprofloxacin, Polystyrene nanocomposite, modifications, adsorption, characterizations

## Abstract

There are different ways for antibiotics to enter the aquatic environment, with wastewater treatment plants (WWTP) considered to be one of the main points of entrance. Even treated wastewater effluent can contain antibiotics, since WWTP cannot eliminate the presence of antibiotics. Therefore, adsorption can be a sustainable option, compared to other tertiary treatments. In this direction, a versatile synthesis of poly(styrene-block-acrylic acid) diblock copolymer/Fe_3_O_4_ magnetic nanocomposite (abbreviated as P(St-*b*-AAc)/Fe_3_O_4_)) was achieved for environmental applications, and particularly for the removal of antibiotic compounds. For this reason, the synthesis of the P(St-*b*-AAc) diblock copolymer was conducted with a reversible addition fragmentation transfer (RAFT) method. Monodisperse superparamagnetic nanocomposite with carboxylic acid groups of acrylic acid was adsorbed on the surface of Fe_3_O_4_ nanoparticles. The nanocomposites were characterized with scanning electron microscopy (SEM), X-ray diffraction (XRD) and vibrating sample magnetometer (VSM) analysis. Then, the nanoparticles were applied to remove ciprofloxacin (antibiotic drug compound) from aqueous solutions. The effects of various parameters, such as initial drug concentration, solution pH, adsorbent dosage, and contact time on the process were extensively studied. Operational parameters and their efficacy in the removal of Ciprofloxacin were studied. Kinetic and adsorption isothermal studies were also carried out. The maximum removal efficiency of ciprofloxacin (97.5%) was found at an initial concentration of 5 mg/L, pH 7, adsorbent’s dosage 2 mg/L, contact time equal to 37.5 min. The initial concentration of antibiotic and the dose of the adsorbent presented the highest impact on efficiency. The adsorption of ciprofloxacin was better fitted to Langmuir isotherm (R^2^ = 0.9995), while the kinetics were better fitted to second-order kinetic equation (R^2^ = 0.9973).

## 1. Introduction

Aside from the well-known pollutants and contaminants in the aquatic environment, compounds of emerging concern (CECs) may impact aquatic life even in very low concentrations [1]. Wastewater influents and effluents can contain CECs, due to their presence in everyday products, such as detergents, fabric coatings, pharmaceuticals, cosmetics, beverages and food packaging [2]. Pharmaceuticals are being detected in drinking and surface water, and although not very persistent, the continuous re-entering increases their abundance, and renders them pseudo-persistent [3]. Pharmaceuticals, include diverse types of compounds, e.g., antibiotics and show low biodegradability. CECs cannot be removed completely by wastewater treatment plants (WWTPs) [2], since WWTP were not designed to treat CECS. In some cases, even less than 10% of CECs is removed, making WWTP effluents a major factor for introducing CECs into the environment [2]. Recently, great attention is given to adsorption technique [4,5,6,7,8,9,10,11,12,13,14,15,16,17,18,19], which is easily applied to the last stage of wastewater treatment plants (WWTPs), with the aim of removing all residues that were not separated and removed from the previous stages.

In particular, special attention is given to find appropriate ways to effectively treat antibiotics from effluents, due to their strong resistance to various decontamination techniques [20]. Available statistics indicate that 100–200 tn of antibiotics are used annually worldwide. As a result, the risk of water resources contamination by these compounds is very high. The residue of those antibiotics in the form of major constituents or metabolites has also been observed in WWTP.

It is noteworthy to mention that the inability of WWTP to remove antibiotics leads to the discharge of those compounds into surface water and underground waters. The inadequate and incorrect use of those compounds, and their continuous entry into the environment, leads to biodistribution and faulty resistance [21,22]. Of the large antibiotic classes, fluoroquinolones are worth mentioning. Antibiotics in this family include Ciprofloxacin (CIP), epinephrine, and norfloxacin. The presence of fluorine atoms in combination with these antibiotics makes these compounds particularly stable, so they are considered to be very dangerous and toxic pollutants in the environment. CIP is detected in sewage and surface water in medical effluents and pharmaceutical plants. The antibiotic can be adsorbed into the sludge and, if applied as fertilizer, it is accumulated in the soil and enters into plants [23]. CIP was observed in surface waters and wastewaters at concentrations below 1 μg/L, while in medical wastewaters in 150 μg/L. Therefore, it is mandatory to find and apply an efficient method for ciprofloxacin removal.

The most important methods used to remove and separate the drug compounds from water and sewage include ozonation, nanofiltration, electron radiation, ion exchange, chemical coagulation and photocatalytic oxidation, all of which have high performance and operation costs [24,25,26,27,28,29]. Nowadays, nanotechnologies are mainly used in water and wastewater treatment, using materials like iron nanoparticles, zeolites and magnetic nanomaterials [30,31]. Among the various methods, adsorption is a simple, environmental friendly, fast, highly efficient and low-cost solution, making it one of the most favorable methods [32,33,34,35,36,37]. 

The removal of pharmaceuticals by adsorption has been the focus of many studies. So far, as adsorptive materials, activated carbon [38,39,40,41] or zeolites [42,43] have been widely used in wastewater treatment. The removal of pharmaceuticals by adsorption shows great potential, due to its easy application into existing water treatment processes. On the other hand, issues regarding adsorbent stability and regeneration costs lead to R&D of innovative and effective adsorbents from polymeric materials. Adsorption processes, such as activated carbon-based have high capital cost, and ineffectiveness and non-selectivity against vat and disperse dyes. Furthermore, saturated carbon regeneration is expensive and leads to adsorbent loss. Depending on the demand, cost, and the nature of the pollutant to be adsorbed, the adsorbents are either disposed or regenerated for future use. The regeneration process of adsorbents needs to be cheap and environmental friendly by recovering valuable adsorbates while reducing the need of virgin adsorbents.

In this study, a versatile synthesis of poly(styrene-block-acrylic acid) diblock copolymer/Fe_3_O_4_ magnetic nanocomposite (abbreviated as P(St-*b*-AAc)/Fe_3_O_4_)) was achieved for environmental applications with a focus on the removal of ciprofloxacin. The nanocomposites were characterized with SEM, XRD and VSM analysis. The nanoparticles were then applied to remove ciprofloxacin (antibiotic drug compound) from aqueous solutions, evaluating the effect of certain important parameters such as the solution’s pH, initial ciprofloxacin concentration, adsorbent dosage and contact time.

## 2. Materials and Methods

### 2.1. Materials

To begin, 4-cyano-4-[(phenylcarbothioyl) sulfanyl] pentanoic acid, as a RAFT agent, was synthesized [32]. Acrylic acid (AAc), styrene (St) monomers, 2, 2-azobisisobutyronitrile (AIBN), and dimethylformamide (DMF), FeCl_2_‧4H_2_O, 99% and FeCl_3_‧6H_2_O, 98% were purchased from Merck (Darmstadt, Germany).

**Figure 1:**
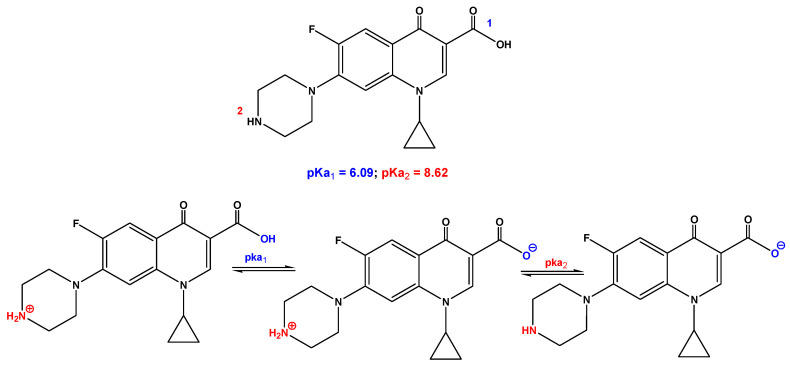
Chemical structure of CIP and its ionizable forms.

The antibiotic model compound used in the present study is ciprofloxacin, purchased from Merck (Germany). Its molecular structure is presented in Figure 1. When it comes to ciprofloxacin’s dissociation and isoelectric constants, the isoelectric point has a value of pI = 7.14, which is calculated by the average of pKa_1_  =  6.09 and pKa_2_  =  8.62. This portrays the two ionizable functional groups of ciprofloxacin; the 6-carboxylic group and the N-4 of the piperazine substituent. pKa1 corresponds to the dissociation of a proton from the carboxyl group, and pKa_2_ corresponds to the dissociation of a proton from the N-4 in the piperazinyl group [44].

### 2.2. Synthesis of Poly(styrene) Homopolymer

RAFT agents (10 mg, 0.036 mmol), styrene monomer (4 mL, 34.96 mmol) and AIBN (3.0 mg, mmol) were added in a 100-mL flask; the reaction was achieved with three freeze pump-thaw cycles under a nitrogen atmosphere. The solution was put to an oil-bath with a temperature of 75 °C for 24 h. The flask was then quenched by cooling. The polystyrene homopolymer was precipitated in methanol. Finally, drying of the product under vacuum at 25 °C for 24 h took place [22].

### 2.3. Synthesis of Poly(styrene-block-acrylic acid), Sphere Superparamagnetic Iron Oxide Nanoparticles (SPIONs) and Poly(St-b-AAc)/Fe_3_O_4_ Supermagnetic Nanocomposite

**Figure 2:**
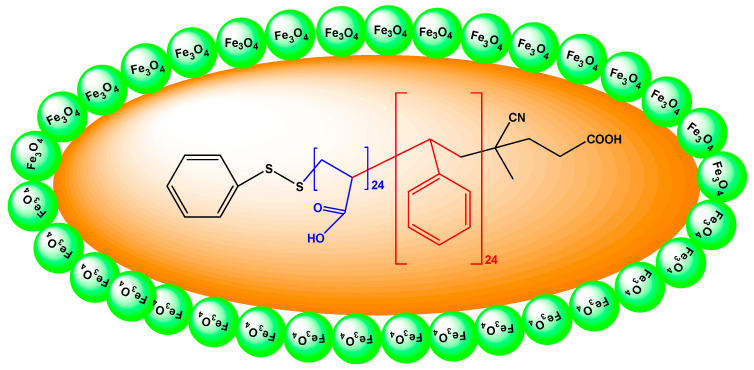
Structure of the prepared poly(St-*b*-AAc)/Fe_3_O_4_.

Macro-RAFT agent (PSt, 200 mg, 19.8 mmol), AAc monomer (1.56 mL, 28.24 mmol), AIBN (3 mg, mmol) and DMF (10 mL) were charged in a two-neck reactor. The reaction was induced using three freeze pump-thaw cycles under a nitrogen atmosphere. The reaction solution was put to an oil-bath with a temperature of 75 °C for 24 h. The reaction mixture was then precipitated in cold diethyl ether (150 mL) and dried under vacuum at 25 °C. The SPIONs were synthesized using a co-precipitation method, as described in literature [22,45]. The poly(St-*b*-AAc)/Fe_3_O_4_ supermagnetic nanocomposite was synthesized as described in a previous study [46]. The final product is magnetic nanocomposite [22] and its structure is illustrated in Figure 2.

It merits clarification that the objective of using magnetic nanoadsorbents and not common adsorbents was the easier separation of solid adsorbent particles from the solution at the end of the process. Due to magnetic particles, using an external magnetic field, poly(St-*b*-AAc)/Fe_3_O_4_ was easily and fast separated from the aqueous solution after adsorption experiments. Also, the preparation of the polysterene nanocomposites was possible (instead of single magnetic particles Fe_3_O_4_), because it contains functional groups which increase its adsorption capacity.

### 2.4. Characterization of Nanoadsorbents

For the XRD patterns, a Bruker XRD diffractometer (Billerica, MA, USA) with CuKα radiation was used. SEM (model Mira 3XMU, TESCAN company, Brno, Czech Republic) was used to study the morphology of nanoparticles.

### 2.5. Preparation of CIP Solutions

Ciprofloxacin hydrochloride (purity 99.8%) was purchased from Alborz Pharmaceutical Company of Qazvin (Qazvin, Iran), and used to prepare the stock CIP solution (100 mg/L prepared with the fixed pre-weighted amount of CIP and the respective volume of Milli-Q ultra-pure water). The residual concentration of CIP after the adsorption experiments was analyzed by (using a) UV-vis spectrophotometer (model Hach DR5000, Duesseldorf, Germany). The concentration of CIP was measured based on previous studies at a wavelength of λ_max_ = 274 nm [47].

### 2.6. Adsorption Experimental Design Method and Data Analysis

In this study, the 7.0.1 Design Expert software was used to determine the number of experiments and the amount of parameters, and to perform the final analysis of the data obtained after the process (Table 1). The measurement of the level of pollutant removal was carried out with the standard design of the statistical model of the CCD (RSM). The main parameters affecting the process are: the initial pH of the medium in the range of 4 to 10, the amount of nanoparticles used in the reaction of 1 to 3 mg/L, the initial concentration of antibiotic ranging from 5 to 25 mg/L, and the reaction time (15 to 60 min).

After the determination of optimal conditions and modeling of the process, the rate of CIP removal was investigated. Finally, the process efficiency in CIP removal was determined using the following equation. The removal (R, %) was also calculated based on the following formula:

(1) $$\mathrm{Removal}=\left(\frac{C_{0}-C_{f}}{C_{0}}\right)\times 100\%.$$

In this relation, R is the efficiency, C_0_ (mg/L) is the initial concentration of CIP, and C_f_ (mg/L) denotes the CIP concentration at the time of t. The amount of adsorbed CIP at equilibrium Q_e_ (mg/g) was calculated from the following equation. In this relation, C_0_ (mg/L)is the initial concentration of CIP, C_e_ (mg/L) denotes the CIP concentration at the time of t, m (g) is the adsorbent mass, and V (L) is the sample volume:

(2) $$Q_{e}=\frac{\left(C_{0}-C_{e}\right)V}{m}.$$

## 3. Results

### 3.1. Characterizations

**Figure 3:**
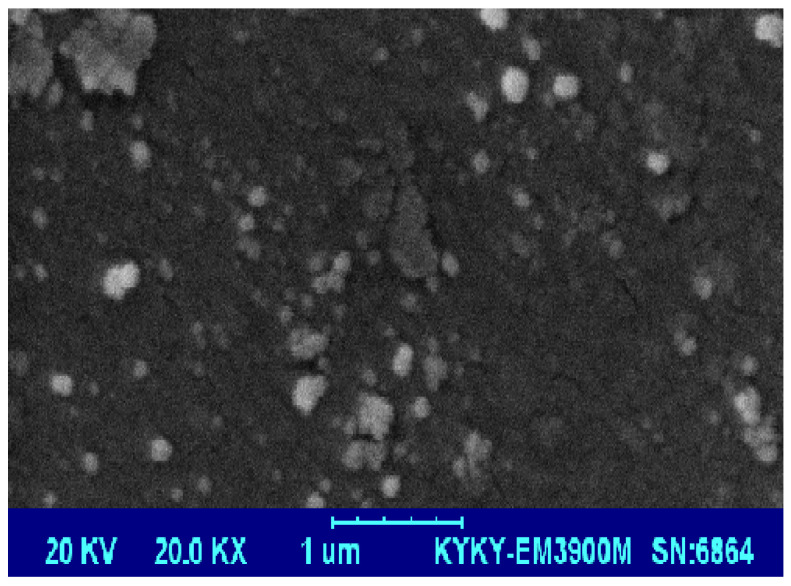
SEM image of P(St-*b*-AAc)/Fe_3_O_4_ nanocomposite.

The morphologies of the P(St-*b*-AAc)/Fe_3_O_4_ nanocomposite are spherical, with D_average_ of 30 nm (Figure 3). It is obvious that the size of spheres is not the same for all particles, due to possible aggregation, but the uniformity regarding the shape is almost the same (spherical).

**Figure 4:**
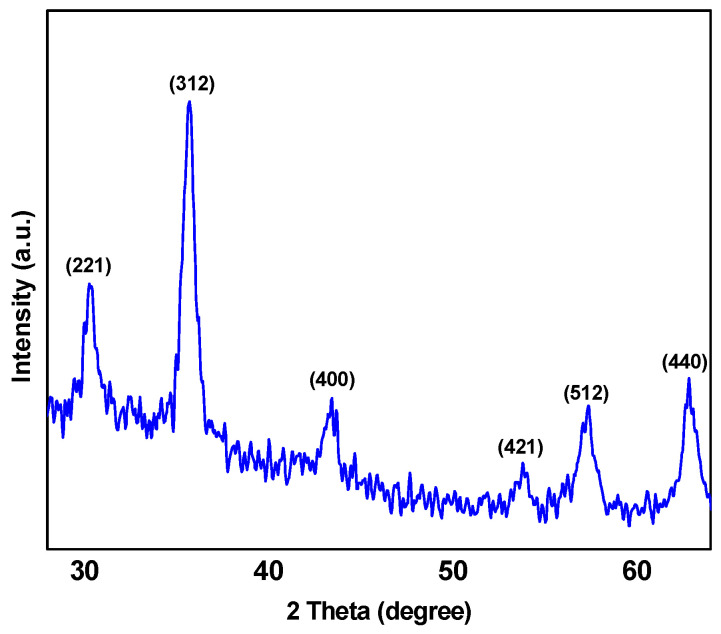
XRD patterns P(St-*b*-AAc)/Fe_3_O_4_ magnetic nanocomposite.

The X-ray diffraction patterns (XRD) resulting from the P(St-*b*-AAc)/Fe_3_O_4_ superparamagnetic nanocomposite are indicated in Figure 4. The resulting peaks at 2θ equal to 30.28, 35.48, 43, 53.4, 57.16, and 63.04° correspond to (221), (312), (400), (421), (512), and (440) prisms of P(St-*b*-AAc)/Fe_3_O_4_ nanocomposite crystalline structure, respectively (Figure 4) [22].

**Figure 5:**
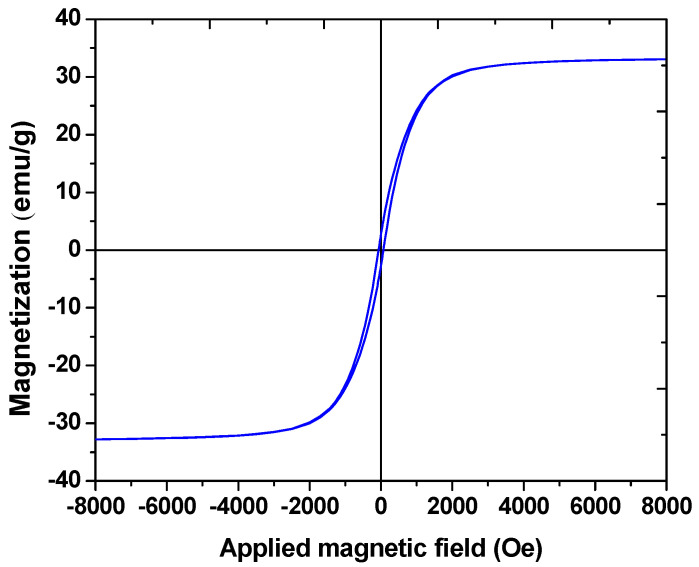
Magnetization curve of P(St-*b*-AAc)/Fe_3_O_4_ supermagnetic nanocomposite.

The super paramagnetic behavior is demonstrated in Figure 5 with a VSM plot. The saturation magnetization of the P(St-*b*-AAc)/Fe_3_O_4_ supermagnetic nanocomposite was around 26 emu/g, which shows that the synthesized magnetic nanocomposite is superparamagnetic.

**Figure 6:**
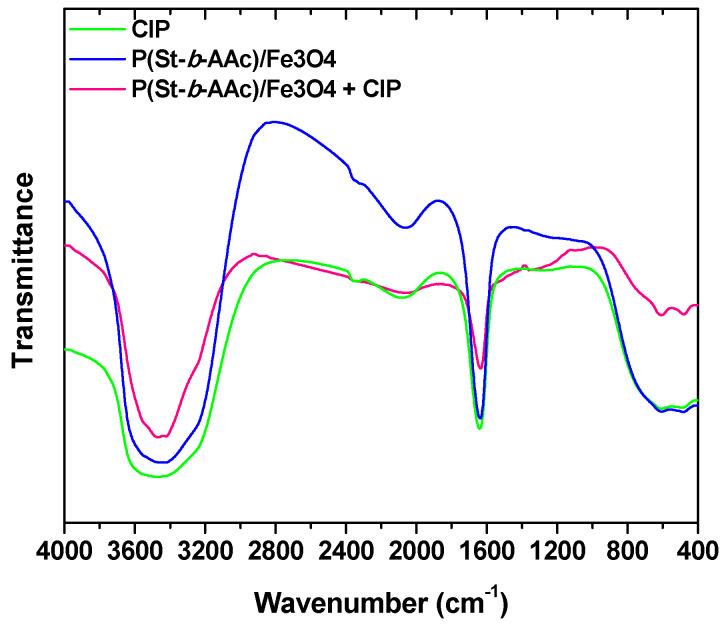
FTIR spectra of CIP, and P(St-*b*-AAc)/Fe_3_O_4_ (before and after adsorption).

A Fourier-transform infrared spectroscopy of nanocomposites was conducted both prior and after adsorption of CIP, and the spectra are presented in Figure 6. Regarding the FTIR spectrum of CIP, a band around 3400 cm^−1^ represent the vibrational frequency of stretching of the N–H bond of the imino moiety on the piperazine group of CIP. Absorption bands at 1633 cm^−1^ and 1080 cm^−1^ represent a primary amine (N–H) bend of the pyridone moiety and the C–F functional group, respectively. On the other hand, the FTIR spectrum related to the CIP-adsorbed nanoadsorbent is, in turn, related to the addition of the nanocomposite to the CIP solution. The broad peaks at 3463 cm^−1^ are attributed to the stretching vibration of O–H bonds. O–H bonds were weaker and shifted down in the presence of ferrite nanoparticles. Similarly, the slight shift at around 1641 cm^−1^ may be related to the interaction of carboxylic groups of polymer with the amino group of CIP (Figure 6). Also, by comparing the FTIR spectra, the intensity of the peaks after adsorption has increased in comparison to those before adsorption, due to the presence of ferrite nanostructures in the CIP solution. 

### 3.2. Data Analysis

For the efficacy evaluation of antibiotic removal of ciprofloxacin using composite P(St-*b*-AAc), a composite design (one of the response surface methods) was used, and the effects of initial antibiotic concentration parameters, pH, adsorbance dose and reaction time were investigated. The response rate is presented in Table 2. The validity of the presented models was analyzed by ANOVA.

In Table 3, the parameters A, B, C and D are the main effect of independent variables, which are the initial concentration of ciprofloxacin, pH, adsorbent dose, and contact time, respectively. The variable AB represents the effect of the initial concentration of ciprofloxacin (factor A) and pH (factor B), and variable A^2^ represents the square effect of factor A on the desired response. 

The proposed model is presented as a modified model by removing non-significant variables via preserving the main effects of variables from the model for the antibiotic elimination efficacy in the following equation:

(3) $$Y\left(\%\right)=67.11-9.43X_{1}-4.7X_{2}+6.26X_{3}+3.71X_{4}-3.9X_{2}{}^{2}$$

In this regard, X_1_, X_2_, X_3_ and X_4_ are coded values of the initial concentrations of antibiotics, pH, adsorbent dose and reaction time. The linear regression is another test that was used to validate the model [48]. In this test, the coefficient of determination (R^2^ = 0.8753), the adjusted coefficient of determination (R^2^_adj_ = 0.8493) and the prediction coefficient (R^2^_pred_ = 0.7955) were calculated and reported. Also, in each model, there is very little difference between the values of R^2^, R^2^_adj_ and R^2^_pred_ is observed.

#### The Effect of Variables on the Process

**Figure 7:**
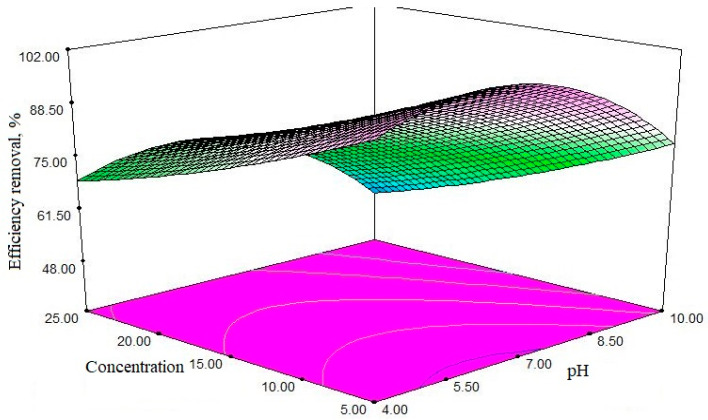
The simultaneous effect of two variables; initial concentration of antibiotic and pH of solution; adsorbent dose of 2 mg/L and reaction time of 37.5 min.

**Figure 8:**
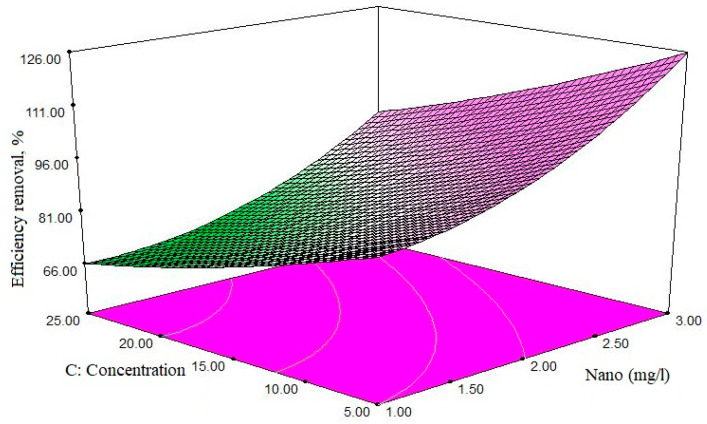
The simultaneous effect of two primary antibiotic and adsorbent dose variables: pH = 7 and reaction time of 37.5 min.

**Figure 9:**
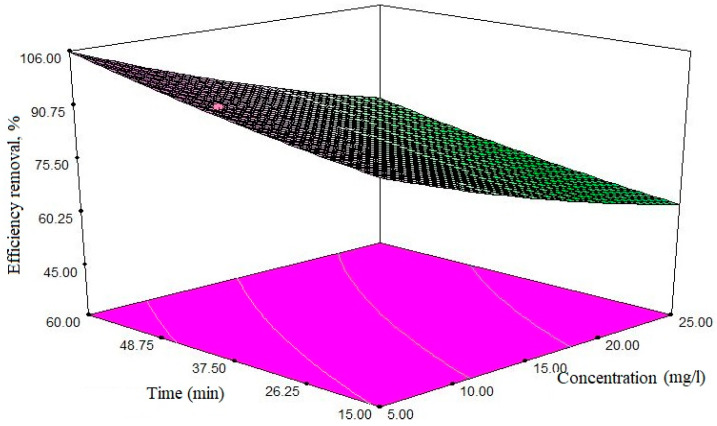
The simultaneous effect of the two initial variables of antibiotic concentration and reaction time: pH = 7 and the adsorbent dose is 2 mg/L.

In order to study the effects of each variable and the interactions or duplicate effects of variables on the response generated by the model, the graphs were based on the polynomial model of the model, using the test design software. According to Equation (3), the initial concentration of antibiotics has the most significant effect on the removal process, with a coefficient equal to 9.94 and the reaction time smallest effect than other parameters with a coefficient of 3.71. The effect of independent variables on the efficacy of antibiotic removal is shown in Figure 7, Figure 8 and Figure 9. Figure 7 shows the effect of the initial concentration of antibiotic and the pH of the solution. As shown from Figure 7, with the increase of antibiotic concentration, the removal efficiency decreases. In particular, with an increase of the antibiotic composition from 16.25 to 25 mg/L, the removal efficiency is reduced from 76.13 to 57.34%, respectively. The ideal efficiency was found to be at pH 6, while at pH > 6 and/or pH < 6, the efficiency is reduced.

The effect of the initial concentration of antibiotics, and the amount of adsorbent, are presented in Figure 8. By increasing the amount of adsorbent effluent, the efficiency increases; for example, when the antibiotic concentration is used at a minimum level and the adsorbent content is 5.5 mg/L, the removal efficiency is approximately 76%. If the concentration is constant and the adsorbent amount is equal to 2 mg/L, the removal efficiency is 95.9%.

The effect of initial CIP concentration and the reaction time are shown in Figure 9. According to the graph, when the antibiotic concentration reaches maximum, and in after 26.5 min, 70.11% of the antibiotic is removed. But when the response time reaches the 2+ level (equal to 48.75 min), removal efficiency is increased to 98.77%.

## 4. Discussion

The contact time is an important factor that directly influences the whole process. In the present work, for a concentration of 5 mg/L, the adsorption process reaches equilibrium at about 37 min, and then shows a relatively stable trend. The effect of the pollutant’s initial concentration is affecting a lot the adsorption process. In this paper, the pollutant’s initial concentration was studied, ranging from 5 to 50 mg/L. As shown in Figure 7, the initial CIP concentration had a negative effect on the elimination efficiency, and by increasing the ciprofloxacin concentration from 16.25 to 25 mg/L, the elimination efficiency decreased from 84 to 57%. The decrease in removal efficiency when increasing initial concentration can be explained by the fact that the active sites are constant with a constant amount of adsorbent dose, but as the concentration of the adsorbent increases, the pollutant molecules (in the medium—water) saturate the available adsorption sites, thereby, the removal efficiency is lowered [49]. Bajpai et al. observed that by increasing the initial concentration of ciprofloxacin from 10 to 20 mg/L, the adsorption capacity increased from 3.74 to 11.32 mg/g [50].

### 4.1. Effect of pH Solution

In the purification processes, including adsorption, pH plays an important role. The Solution’s pH can affect the adsorbent’s surface load, the degree of ionization of various pollutants, the separation of functional groups on active adsorbent sites, as well as the structure of the antibiotic molecule; in effect, the solution’s pH affects the chemical environment of the aqueous and adsorption surface bonds. The pH changes were applied to the range of 4–10, and its effect on the removal efficiency was then analyzed. The removal process had the highest percentage at pH 6.2–7, while with the increase of pH, the removal efficiency decreased.

The effect of pH on the ciprofloxacin molecule has shown that in pH less than 6.2, the surface of the molecule appears cationic and positive due to the protonation of amino groups. At pH values higher than 8.6, the ciprofloxacin molecule is converted into anionic form, due to the loss of the proton from the carboxylic group in the antibiotic structure. In the range of 6.2 to 8.6, the deprotonation of carboxyl groups leads to negative carboxylate production. However, the amino group of proteins has a positive charge. In other words, it has a positive and a negative “head”. The stabilization and behavior of ciprofloxacin molecule from 6.2 to 7.8 have also been investigated [51]. Since the pH value at pH_pzc_ at the isoelectric absorption point is 7.5, and is negatively charged at higher pH values, given that at pH values above 7.5, both the adsorbent and the antibiotic molecule are both negatively charged. At a pH of less than 6.2, the adsorbent and the antibiotic have positive charge, so in this range, the adsorption process occurs slower and reaches at minimum removal rate at pH = 6.2-6.8, because the unnamed bands reach the maximum electrostatic gravity.

### 4.2. Effect of Adsorbent’s Dose

Based on the findings of this study, the adsorbent dose was the most important factor affecting the efficiency of ciprofloxacin elimination. The study of the effect of adsorbent mass on adsorption processes is one of the most important issues to be considered. Adsorption dose was applied to the range of 1 to 3 mg/L, and its effect on the effectiveness of ciprofloxacin antibiotic removal was measured. Depending on the results obtained using constant concentrations of antibiotics, the increase in the dose of the adsorbent improves the removal efficiency. As shown in Figure 8, when the concentration of antibiotic is constant and equal to 16.25 mg/L, and the amount of adsorbent is 1.5 mg/L, the removal efficiency is 75.97%—and when the amount of adsorbent reaches 2 mg/L, the removal efficiency is improved, reaching 95.91%; at a constant concentration of antibiotic, by increasing the dose of adsorbent, the ratio of active sites on the adsorbent’s surface is high relative to the adsorbing molecules (pollutants), resulting in increased elimination efficiency. On the contrary, in low adsorbent amounts, the ratio of active sites to the adsorbent molecules is lower, and the adsorption decreases.

On the other hand, with the increase of adsorbent above the optimal amount, the adsorption capacity decreased below the maximum level of 15.25 mg/g, which is also due to the fact that by increasing the adsorbent dose, the total capacity of the active sites present in the adsorbent level is completely covered. If not, its adsorption capacity is reduced. This can be the use of available surface in the form of unsaturated attributed adsorbent. The results show that the adsorption pattern in the non-saturable adsorbent form causes undesirable use of existing spaces; this issue is very important in the design of the process economics, particularly in scaling-up.

In this study, 5 mg/L of antibiotic and 2 mg/L of adsorbent were introduced as the optimum amount, at maximum efficiency, with application of 2 mg/L of adsorbent, despite the increase in adsorbent content, other increase in cleavage removal efficiency has not shown any increase. In other words, the removal rate remains constant. It can be concluded that this amount of adsorbent adsorbs all the antibiotics in the solution. Therefore, the antibiotic concentration in the solution is so low that it is no longer “able to be adsorbed” easily. A study by Peasant et al. also showed that with the increase in the adsorbent dose (chitosan/zeolite composite), the dye removal increases, due to the increasing number of adsorption sites, while the increase of adsorbent’s dose reduces the adsorption capacity (from the maximum of 17.77 mg/g) [52].

### 4.3. Effect of Contact Time

An important issue when using the adsorption system is providing an effective contact time under specific conditions. In this paper, contact time was applied to the range of 15 to 60 min, and its effect on the ciprofloxacin antibiotic removal. Figure 7 shows that the adsorption process reaches equilibrium at different times. For a concentration of 5 mg/L, the adsorption process reaches equilibrium at about 37 min, and then shows a relatively stable trend. By increasing contact time, the probability of colliding with adsorbent molecules is also increased, and the efficiency of removal increased. Chang et al. (2012) obtained the equilibrium time for tetracycline removal by Monte Myrnolite for 8 h [53]. In another study by Liu et al. who removed tetracycline using zeolite= by increasing contact time, resulted in the removal efficiency also increased, and the time of equilibrium was 120 min [54].

### 4.4. Kinetics and Adsorption Isotherms

The adsorption kinetics depends on the adsorbent chemical and physical properties, which influence the adsorption mechanism. In this study, we have used different kinetic and isotherm adsorption models such as pseudo-first order, pseudo-second order, Langmuir, and Freundlich (Table 4).

**Figure 10:**
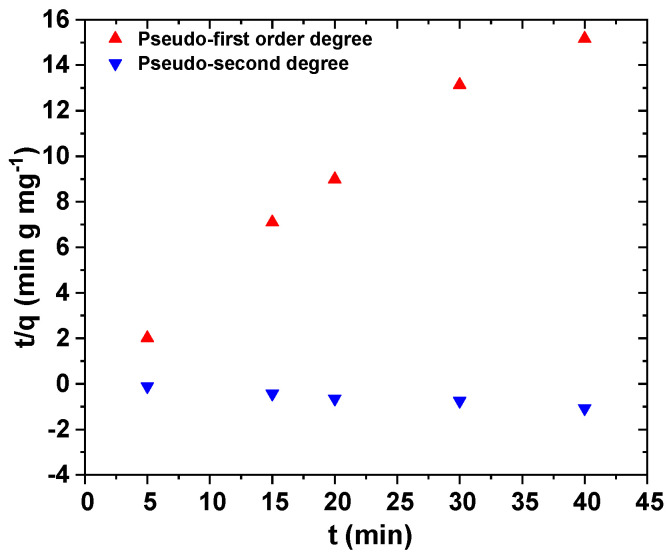
(Up triangle): pseudo-first order kinetic equation; (down triangle) pseudo-second order kinetic equation.

The pseudo-first and pseudo-second order kinetic equations are shown in Figure 10. Adsorption kinetics were used to determine the control mechanism of adsorption processes. Thus, in this figure, the experimental points were not shown, and only theoretical ones are presented. Based on Table 5, the best fitting was achieved with pseudo-second order equation (R^2^ = 0.9984).

**Figure 11:**
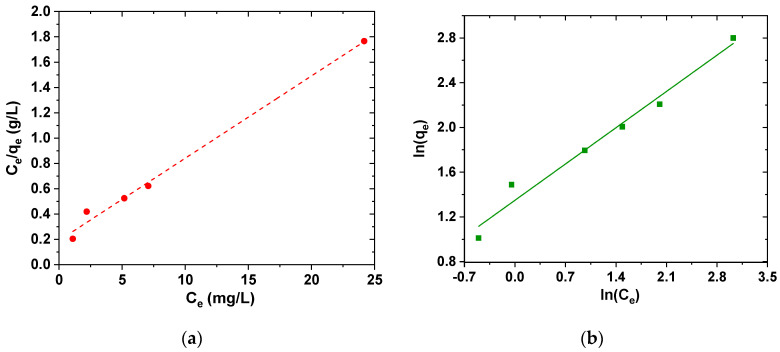
Equilibrium results fitted to (**a**) Langmuir isotherm and (**b**) Freundlich isotherm.

The isotherm of adsorption describes how the adsorbent and adsorbate interact. In this study, the experimental results were fitted to Freundlich and Langmuir isotherms. The Langmuir model is valid for single-layer adsorption on adsorbent surface, with limited and uniform adsorption locations, while the Freundlich isotherm is based on single-layer adsorption on heterogeneous adsorption sites with unequal and non-uniform energies. Figure 11 shows the relative isotherms.

In Freundlich isotherm, when K_F_ increases, the adsorbent material adsorbed higher amounts of pollutant, and the value of n between 1 and 10 reflects the proper adsorption process. The parameters and coefficients are briefly summarized in Table 5. In this study, the calculated K_F_ value is 4.75, and the value of n is 2.79, which is within the specified range. Therefore, the adsorption of ciprofloxacin on the adsorbent is well fitted to Langmuir model (Table 6), but it is fact that the data may suggest the presence of non-specific or multi-type interactions between the adsorbate molecules and the adsorptive sites.

**Figure 12:**
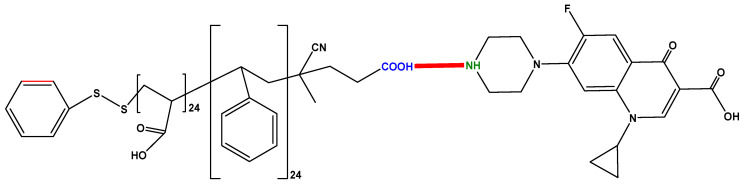
Possible adsorption interaction.

A major concern regarding any synthesized adsorbent material is answering why this material was synthesized instead of another structure-type material? To respond, it is of fundamental importance to mention some facts. Nanoparticles have a unique combination of properties, such as small size, large surface area, catalytic potential, large number of active sites, high chemical reactivity; all of the above give nanoparticles high adsorption capacity [59]. Also, magnetic nanoadsorbents can be applied as cost-saving and effective materials to separate the materials (solid) from the liquid-phase (water) after the end of the adsorption process. Moreover, the relatively simple isolation of magnetic materials from the solution can aid to their regeneration and reuse [60]. Therefore, the magnetic nanoadsorbents can be good candidates for water/wastewater treatment. Based on the above, Poly(vinylimidazole-co-divinylbenzene) magnetic nanoparticles have been used for the adsorption of fluoroquinolones from aqueous environments [61]. Wang et al. also synthesized the easy to separate magnetic chalcogenide composite KMS-1/L-Cystein/Fe_3_O_4_ using L-cystein to connect KMS-1 and Fe_3_O_4_ nanoparticles for ciprofloxacin removal from aqueous solutions [62]. Table 7 shows a brief comparison of some other adsorbent materials tested for the removal of CIP. However, similar experimental conditions should be kept in order to compare two adsorbents (even for the treatment of the same pollutant). Parameters affecting adsorption are the contact time, the solutions’ pH, the initial concentration of the pollutant, temperature, adsorbate volume, agitation speed, the solution’s ionic strength, and adsorbent dosage. Any change to the abovementioned conditions will lead to different results, and the comparison can be made for adsorbent/adsorbate systems of the same study. Also, based on the interaction groups, a possible mechanism of adsorption is illustrated in Figure 12.

### 4.5. Aspects

It is known that activated carbon is a very popular adsorbent material, with the demand for virgin activated carbon expanding, since demand from water and wastewater treatment facilities has been steadily increasing. Together with the increase of wastewater treatment applications, the demand and production of activated carbon is also increasing. The largest quantities of activated carbon consumption are observed the U.S.A, Japan and then Europe [72]. Antibiotics are being detected in the aquatic environment. There are different ways for antibiotics to enter the aquatic environment with WWTP considered to be one of the main points of entrance. Even treated wastewater effluent can contain antibiotics, since WWTP cannot eliminate the presence of antibiotics. Compared to other tertiary treatments, adsorption can be a sustainable option for antibiotic removal from wastewaters. Activated carbon is used in the pharmaceutical for the removal of unwanted compounds [72]. Activated carbon possesses a plethora of disadvantages [73], such as high capital cost, ineffectiveness and non-selectivity against vat/disperse dyes. Furthermore, saturated carbon regeneration is expensive and leads to adsorbent loss. Depending on the demand, cost, and the nature of the pollutant to be adsorbed, the adsorbents are either disposed or regenerated for future use. Used adsorbents are considered hazardous waste, causing environmental and societal problems in various countries [74]. Heat accumulation and toxic adsorbates desorption could create hazardous conditions. In addition, odor can be caused by the dumping of adsorbents. 

Since regeneration costs can be quite high, the reduction of consumption costs is the key to sustainable and industrial benefits. Substantial studies regarding the activated carbon-based adsorption of pollutants onto have been conducted, but research on regeneration methodologies remains limited [75]. Adsorbent regeneration capability cost analysis is necessary for the economic and environmental assessment of the adsorption process. For the spent adsorbent stabilizing or proper disposal seem to be difficult. The regeneration process of adsorbents from the points of view of sustainability and the environmental involves recovering valuable adsorbates, while reducing the need of virgin adsorbents, and this is extremely important. Studies on novel adsorbents, at full-scale adsorption systems, should be considered for potential industrial applications. 

## 5. Conclusions

Antibiotics are still being detected in the effluents of WWTP, and adsorption seems to be a sustainable option for antibiotics removal from waters. Poly(St-*b*-AAc) diblock copolymers were prepared using the RAFT technique. This copolymer with acrylic acid group was adsorbed onto the surface of Fe_3_O_4_ nanoparticles, through the interaction with hydroxyl groups on the Fe_3_O_4_ nanoparticles’ surface. A magnetic nanocomposite ranged in 30 nm was then prepared. The VSM analysis showed the saturation magnetization (26 emu/g for P(St-*b*-AAc)/Fe_3_O_4_). The removal process was performed using P(St-*b*-AAc)/Fe_3_O_4_ to remove ciprofloxacin antibiotic from synthetic sewage. The effects of parameters such as initial concentration of antibiotic, pH, soluble dose and reaction time were studied. The primary concentration of antibiotics with the highest negative effect and adsorbent dose showed the most positive effect in the removal process. The results also indicated that 97.5% of antibiotics were removed under optimal conditions, which include an initial antibiotic concentration of 5 mg/L, pH 7, and an adsorbent dose of 2 mg/L for 37.5 min. The adsorption of CIP was better fitted to Langmuir isotherm (R^2^ = 0.9995), while the kinetics were better fitted to second-order kinetic equation (R^2^ = 0.9973). Future work should include multi-component pharmaceutical adsorption with continuous adsorption of wastewaters, taking into account adsorbent regeneration.

## Figures and Tables

**Table 1:** Design parameters together with the values and regions selected.

Parameters	Level of Parameters		
	−α	0	+α
A: pH	4	7	10
B: Mass (mg)	1	2	3
C: Concentration (mg/L)	5	15	25
D: Reaction time (min)	15	37.5	60

**Table 2:** Operational parameters and their efficacy in the removal of Ciprofloxacin antibiotic in different methods of performing runs.

Removal (%)	D (Time (min))	C (Nano(g))	B (pH)	A (Initial Concentration)	Run
73.11	1	1	1	−1	1
62.45	0	0	0	0	2
79.04	1	1	1	−1	3
99.5	0	0	0	−2	4
71.06	0	0	0	0	5
65.45	0	0	0	0	6
62	1	−1	0	1	7
89.73	0	2	1	0	8
60.91	1	1	1	1	9
57.03	−1	1	1	1	10
45.76	−1	−1	1	1	11
55.32	1	−1	1	1	12
66.7	−1	1	−1	1	13
66.6	0	0	0	0	14
72.8	1	−1	1	−1	15
53.68	−1	−1	−1	1	16
58.52	−1	−1	1	−1	17
67.5	−1	1	1	−1	18
57.74	−2	0	0	0	19
81.32	−1	1	−1	−1	20
56.45	0	0	−2	0	21
45.2	0	0	0	2	22
67.51	0	0	0	0	23
70.75	0	0	0	0	24
76.63	1	−1	−1	−1	25
51.12	0	−2	0	0	26
40.65	0	0	2	0	27
71.59	2	0	0	0	28
84.24	1	1	−1	−1	29
72.2	−1	−1	−1	−1	30

**Table 3:** ANOVA for Response Surface Quadratic Model.

Source	Sum of Squares	*p* Value	*F* Value	Mean Square	df	
		Prob > *F*				
Model	4492.14	0.0001	8.06	320.87	14	
A-Nano	353.88	0.0093	8.89	353.88	1	significant
B-pH	113.35	0.1121	2.85	113.35	1	
C-Concentration	822.02	0.0004	20.66	822.02	1	
D-Time	70.22	0.2039	1.76	70.22	1	
AB	1.32	0.8579	0.033	1.32	1	
AC	27.36	0.4199	0.69	27.36	1	
AD	12.83	0.5785	0.32	12.83	1	
BC	4.88	0.731	0.12	4.88	1	
BD	16.16	0.5335	0.41	16.16	1	
CD	1	0.8762	0.025	1	1	
A^2	159.99	0.0633	4.02	159.99	1	
B^2	318.55	0.0127	8.01	318.55	1	
C^2	150.96	0.0704	3.79	150.96	1	
D^2	2.42	0.8086	0.061	2.42	1	
Residual	596.83			39.79	15	
Lack of fit	541.99	0.0459	4.94	54.2	10	
Pure Error	54.83			10.97	5	significant
Cor. Total	5088.97				29	

**Table 4:** Equations used in this study.

Equation	Expression
Pseudo-first-order [55]	$\log(q_{e}-q_{t})=\log\left(q_{e}\right)-t\frac{k_{1}}{2.303}$ q_e_ is the amount of mass absorbed in equilibrium state (mg/g) q_t_ is equal to (mg/g) the amount of mass absorbed at time t k_1_ is the equilibrium of the first-order kinetic velocity (min^−1^)
Pseudo-second-order [56]	$\frac{t}{q_{t}}=\frac{1}{k_{2}q_{e}{}^{2}}+t\frac{1}{q_{e}}$ k_2_ is the constant of the equilibrium velocity of the quadratic kinetic equation (g mg^−1^ min^−1^)
Freundlich [57]	$\ln\left(q_{e}\right)=\ln\left(K_{F}\right)+\frac{1}{n}\ln\left(C_{e}\right)$ C_e_ is the equilibrium concentration in the solution after adsorption (mg/L) n and K_F_ are the Freundlich constants
Langmuir [58]	$\frac{C_{e}}{q_{e}}=\frac{1}{q_{m}b}+\frac{C_{e}}{q_{m}}$ q_m_ represents absorption capacity (mg/g) b is the Langmuir constant (L/mg)

**Table 5:** Parameters and related kinetic coefficients.

Kinetic Constant Rate	R^2^	Kinetic Model
0.0320 min^−1^	0.7862	Pseudo-first order
1.91 g mg^−1^ min^−1^	0.9984	Pseudo-second order

**Table 6:** Parameters and correlation coefficients of isotherm models.

q_m_ = 15.52 mg/g	b = 0.689 L/mg	R^2^ = 0.9918	Langmuir isotherm
K_F_ = 4.75 (mg^1-n^ L^n^/g)	n = 2.79	R^2^ = 0.9845	Freundlich isotherm

**Table 7:** CIP adsorption capacities comparison from aqueous solutions using various adsorbents.

Adsorbent	Q_m_ (mg/g)	Reference
Carbon nanotubes	135	[29]
Kaolinite	6.99	[29]
Bamboo-based carbon modified	153.17	[63]
Graphene oxide	379	[64]
Ca^2+^-montmorillonite	330	[65]
Multi-walled nanotubes	194	[66]
Iron hydrous oxide	25.76	[67]
Aluminum hydrous oxide	14.72	[67]
Bentonite	147	[68]
Birnessite	80.96	[69]
Montmorillonite	137.7	[70]
Al-PILC	17.78	[71]
Polystyrene nanocomposites P(St-*b*-AAc)/Fe_3_O_4_	15.52	This study
